# Ultrasonographic Study of Gallbladder Wall Thickness and Emptying in Cirrhotic Patients without Gallstones

**DOI:** 10.1155/2009/683040

**Published:** 2009-08-10

**Authors:** Massimiliano Loreno, Salvatore Travali, Anna Maria Bucceri, Giuseppe Scalisi, Carla Virgilio, Alfio Brogna

**Affiliations:** ^1^Department of Internal Medicine and Internal Specialities, University of Catania, Via S. Sofia n. 86, 95100 Catania, Italy; ^2^Department of Biomedical Sciences, Section of Clinical Pathology and Molecular Oncology, University of Catania, Via Androne 87, 95124 Catania, Italy

## Abstract

*Background and Aim*. Gallbladder wall thickening and impaired contractility are currently reported in cirrhotic patients and often related to portal hypertension and hepatic failure. The purpose of this work was to evaluate, by ultrasonographic method, gallbladder wall thickness and gallbladder emptying after a standard meal in normal subjects and in patients with compensated liver cirrhosis without gallstones. *Methods*. Twenty-three patients with Child-Pugh class A liver cirrhosis and twenty healthy controls were studied. Gallbladder wall thickness (GWT), gallbladder fasting volume (FV), residual volume (RV), and maximum percentage of emptying (%E) were calculated. Measurements of mean portal velocity, portal vein flow, and serum albumin were performed too. Statistical analysis was assessed by Student's “*t* test” for unpaired data. *Results*. GWT was 0.60 ± 0.22 cm in cirrhotic patients and 0.21 ± 0.06 cm in controls (*P* < .0001). FV and RV were, respectively, 37.8 ± 3.7 cm^3^ and 21.8 ± 3 cm^3^ in cirrhotic patients, 21.9 ± 4.2 cm^3^ and 4.6 ± 2.2 cm^3^ in healthy volunteers (*P* < .0001). %E was smaller in cirrhotics (42.6 ± 7.8) as compared to controls (80.3 ± 7.2; *P* < .0001). *Conclusions*. In patients with compensated liver cirrhosis without gallstones gallbladder wall thickness is increased whereas its contractility is reduced. These early structural and functional alterations could play a role in gallstone formation in more advanced stages of the disease.

## 1. Introduction

Gallbladder wall thickening and impaired contractility are currently reported in cirrhotic patients, but most published studies were carried out in patients with portal hypertension and hepatic failure [1–3].

Diffuse gallbladder wall thickening is a nonspecific alteration caused by several disorders, including both intrinsic diseases (acute cholecystitis and gallbladder carcinoma) and extracholecystic diseases, such as acute hepatitis, liver cirrhosis, hypoalbuminemia, congestive heart failure, acquired immunodeficiency syndrome, pancreatitis, myeloma, and acute pyelonephritis. 

Gallbladder emptying in response to a meal is a physiological phenomenon, mainly coordinated by the rate of gastric emptying of foods in duodenum and by the subsequent release of cholecystokinin (CCK), which triggers, gallbladder contraction. In normal subjects gallbladder emptying is affected by several factors: age, body surface area, wall thickness, fasting volume, hormonal factors, and meal composition. Impaired gallbladder contractility has been suggested to increase the incidence of gallstones in cirrhotic patients, although incongruous results are reported [4, 5].

Real-time ultrasound (US) is the method used for direct gallbladder visualization under physiological and pathological conditions, since it allows repeated measurements at short intervals and provides information for the study of gallbladder wall thickness, content, and contraction. Ultrasound assessment of spleen dimensions and portal vein diameter is a useful noninvasive method able to lead to the diagnosis of portal hypertension. Moreover, echodoppler flowmetry is able to quantitatively assess portal flow and mean blood velocity. A significant correlation between the reduction in portal flow velocity and the severity of the disease, evaluated by the Child-Pugh score is reported in literature [6]. 

The purpose of our study was to evaluate, by ultrasonographic method, gallbladder wall thickness and emptying after a standard meal in normal subjects and in patients with Child-Pugh class A liver cirrhosis without gallstones and to verify if compensated liver cirrhosis, without signs of portal hypertension, could determine gallbladder wall alterations and/or dysfunctions. 

## 2. Patients and Methods

The study involved 23 consecutive patients (13 males and 10 females; mean age 54.5 years, range 42–67; mean BMI 23.8 kg/m^2^, range 18.2–26) affected by Child-Pugh class A liver cirrhosis without gallstones. The diagnosis of liver cirrhosis was made on the basis of clinical and biochemical features and confirmed by histological examination. Abdominal ultrasound and gastrointestinal endoscopy were performed in order to exclude the presence of splenomegaly, subclinical ascites, gastroesophageal varices, and portal hypertensive gastropathy*.*

The aetiology of cirrhosis was viral in 16 patients (69.5%), alcoholic in 4 patients (17.4%), mixed alcoholic/HBV in 2 patients (8.7%), and autoimmune in 1 patient (4.3%).

Child-Pugh class was assigned on the basis of biochemical and clinical characteristics.

The control group included 20 healthy subjects (10 males and 10 females; mean age 53.6 years, range 43–66; mean BMI 22.8 kg/m^2^, range 18–24.8). 

All subjects included in the study were nonsmokers and they were not taking any medication affecting gastrointestinal motility; none of them had undergone any gastric or ileal surgery, and none was diabetic. 

Local Hospital Institutional Review Board reviewed this protocol and the study conformed to the ethical guidelines of the 1975 Helsinki Declaration. Written informed consent was obtained from each subject.

A single operator performed all the ultrasound examinations both in cirrhotic patients and in controls. Real-time bidimensional and doppler ultrasound examinations were performed using a 3.5 MHz transducer (Logiq P5 Pro, GE Healthcare). Gallbladder was examined by means of the images obtained in both supine and left lateral decubitus in order to evaluate wall thickness, longest axis, width, and depth; portal vein was studied in supine decubitus and suspended respiration.

On the morning of the test, after an overnight fast, basal measurements of gallbladder wall thickness (GWT), portal vein diameter (PD), portal cross-sectional area (CSA), mean portal velocity (PV), portal vein flow (PVF), and gallbladder fasting volume (FV) were taken in all subjects. 

GWT was measured in longitudinal scan with ultrasound beam orthogonally oriented at the level of the gallbladder anterior wall. PD was assessed during normal suspended respiration, measuring the distance between the anterior and posterior wall, at the point where the vein crosses the hepatic artery. The average of the three measurements obtained in longitudinal and transverse scan was accepted as portal diameter. The cross-sectional area was calculated using the formula: 1/4 *π* × PD^2^, assuming the section of the vein to be circular. 

To assess PV, the portal vein was firstly visualized along its longitudinal axis and then a sample volume, half the size of the vessel diameter, was positioned in the middle of the vessel lumen, at the crossing point with the hepatic artery. The Doppler angle was set below 60 degrees and mean flow velocity was automatically calculated by the equipment from the spectral analysis. Pulse repetition frequency and wall filter were set at 4 KHz and 100 Hz, respectively. The values were obtained calculating the average of three measurements of mean portal velocity. Intraobserver variability in bidimensional and doppler measurement was assessed by examining 15 patients and 8 control subjects three times each by the same operator (A.B.). The overall variability was lower than 8%. Gallbladder volume was calculated using the ellipsoid formula V = 1/6 *π*abc, where a = maximum length, b = maximum width, and c = maximum depth [7]. Maximum length and depth were taken after gallbladder visualization in longitudinal plane; maximum width was taken in transverse plane. FV was calculated as average of two measurements taken at 5-minute interval.

Blood was also drawn in order to assess albumin levels in both patients and controls. 

Each subject was then invited to eat, within about 15 minutes, a standard solid 650 Kcal meal consisting of pasta (40 g), beef hamburger (100 g), bread (50 g), salad (200 g), an apple (200 g). The nutritional breakdown of the meal was 19% proteins (30.8 g), 30% lipids (21.6 g), 51% carbohydrates (82.9 g), and 3.2 g of vegetable fibres.

Allowing for the lag phase of solid gastric emptying, gallbladder volume was initially measured sixty minutes after the beginning of the meal (time 0). Further measurements were obtained at 15, 30, 45, 60, 75, 90, 105, 120, 135 minutes and then continued at 15-minute intervals until the end of gallbladder contraction, to make sure the actual residual volume was reached and refilling started. Residual volume (RV, the smallest volume after the test meal) and percentage of emptying (%E = 1 − RV/FV × 100) were calculated.

## 3. Statistical Analysis

GWT was expressed in cm and gallbladder volumes (FV and RV) were expressed in cm^3^. PD was expressed in mm, PV as cm/sec, and PVF as mL/min. Results are expressed in mean ± SD. Differences between groups were analyzed performing two-tailed Student *t* test for unpaired data with statistical significance set at *P* ≤ .05 level.

## 4. Results

Clinical and demographic characteristics of cirrhotic patients and healthy controls are reported in Table 1. Doppler and bidimensional ultrasound measurements are reported in Table 2  and Table 3.

GWT was significantly greater in Child-Pugh class A cirrhotic patients (0.60 ± 0.22 cm) as compared to control subjects (0.21 ± 0.06 cm; *P* < .0001). Albumin levels were 3.03 ± 0.34 g/dL in the cirrhotic group and 3.65 ± 0.29 g/dL in controls (*P* < .0001). In cirrhotic patients mean PD was 8.42 ± 0.87 mm, mean PV was 11.63 ± 1.65 cm/sec and mean PVF was 652 ± 157 mL/min. In control subjects mean PD was 8.01 ± 0.76 mm, mean PV was 12.56 ± 1.55 cm/sec and mean PVF was 642 ± 159 mL/min. No statistically significant difference was observed between the two groups when we compared mean PD (*P* = .1), mean PV (*P* = .06) and mean PVF (*P* = .84).

Mean FV was larger in Child-Pugh class A cirrhotic patients (37.8 ± 3.7 cm^3^) than in controls (21.9 ± 4.2 cm^3^) and the difference was statistically significant (*P* < .0001). 

**Figure 1:**
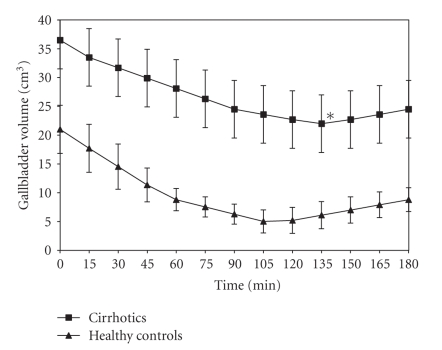
Gallbladder emptying curves after the test meal in Child-Pugh class A cirrhotic patients and in healthy controls (mean ± SD). The asterisk indicates statistical significant difference in cirrhotics (*P* < .0001) versus controls.

Mean RV was reached at 135 minutes in Child-Pugh A cirrhotics and at 110 minutes in healthy controls. RV was significantly larger (21.8 ± 3.0 cm^3^) in cirrhotic patients than in controls (4.6 ± 2.2 cm^3^; *P* < .0001), while maximum %E was smaller in cirrhotics (42.6 ± 7.8) as compared to controls (80.3 ± 7.2; *P* < .0001). Gallbladder emptying curves are reported both in Child-Pugh class A cirrhotic patients and in healthy controls (Figure 1). 

No significant difference in gallbladder volumes and emptying was observed among cirrhotics with chronic alcohol abuse.

## 5. Discussion

In liver cirrhosis, gallbladder wall thickening is frequently observed [1] and it is often reported in association with portal hypertension [2]. In a hamster cirrhosis model, portal hypertension was associated with submucosal oedema and areas of dilated vessels in the gallbladder wall. These histological changes were related to gallbladder wall thickening and associated with impaired wall contractility [8]. In a previous work, we demonstrated significant gallbladder wall thickening in cirrhotic patients with ascites [9]. In the present study, we observed wall thickening in almost all Child-Pugh class A liver cirrhotic patients. These patients lacked clinical signs of portal hypertension: they had no splenomegaly, gastroesophageal varices, or hypertensive gastropathy.

It's well known that portal hypertension has a crucial role in the transition from preclinical to clinical phase of liver cirrhosis. It contributes to the occurrence of ascites and encephalopathy and directly causes the development of collateral circulation and variceal hemorrhage. To date, portal pressure can be measured only by invasive methods and the calculation of hepatic vein pressure gradient (HVPG), with the catheterization of a hepatic vein via the femoral or jugular route, is the most widely used.

The quantitative assessment of portal blood flow, by doppler ultrasound method, is affected by different physiological and hemodynamic conditions. Portal blood flow is usually normal in cirrhotic subject because of enlarged portal caliber [10]. On the other hand, mean portal velocity is often reduced. This parameter has been related to the progression of portal hypertension [11], even though we actually lack an absolute threshold value discriminating between normal subjects and cirrhotic patients. 

In our study, no statistical differences in portal diameter and doppler parameters were found between cirrhotic patients and normal subjects. The same results were observed when we compared different groups of cirrhotic patients, according to the aetiology of liver disease. We therefore assume that, at least in Child-Pugh class A patients, portal hypertension is not the fundamental cause of gallbladder wall thickening. 

Several studies reported that hypoalbuminemia is a major determining factor in gallbladder wall thickening, whereas other studies did not demonstrate such a correlation [12, 13]. Our patients showed reduced albumin levels as compared to normal subjects. Hypoalbuminemia, causing oedema and structural changes of the gallbladder wall, could induce wall thickening and also affect gallbladder contractility. Therefore, the mechanisms responsible for wall thickening seem to be precocious and active in the early stages of liver cirrhosis and precede the onset of portal hypertension. 

Some studies have demonstrated that in subjects without lithiasis, gallbladder volume is increased in the population over the age of 50 years [14], as previously described for the common bile duct [15] and the pancreatic ductal system. An increased gallbladder volume, causing a defect in motility and bile stasis, could play an important role in stone formation. Our study showed that both gallbladder fasting volume and residual volume were significantly increased in Child-Pugh class A cirrhotic patients without gallstones. Probably these changes occur before the onset of clinical and ultrasound signs of portal hypertension. Whereas the existence of higher fasting gallbladder volumes in liver cirrhosis is commonly accepted [16], few results regarding gallbladder emptying have been reported in literature [4, 5]. Our data show that in Child-Pugh class A liver cirrhotic patients the maximum percentage of gallbladder emptying was reduced, suggesting an overall reduction of gallbladder contractility. 

To date, most studies involved patients with signs and symptoms of portal hypertension; in some studies gallbladder hypocontractility was found to be related to the severity of liver cirrhosis [5]. Nevertheless, the cause of the impaired gallbladder emptying remains to be established. Under physiological conditions, the gallbladder contractile activity is primarily regulated by CCK, whose levels are often increased in cirrhosis [17]. On the other hand, patients with cirrhosis show raised serum bile salt concentrations which directly inhibit CCK-induced gallbladder contraction, contributing to the impairment of gallbladder emptying [18]. Gallbladder hypocontractility could be induced by enhanced levels of endogenous nitric oxide, which has a tonic relaxing influence on gallbladder smooth muscle and could cause significant impairment of motility in patients with portal hypertension [19]. 

Autonomic neuropathy, a common disorder in patients with chronic liver disease irrespective of the aetiology, is another factor suggested to be responsible for impaired gallbladder motility [20]. 

Cirrhotic patients have a higher incidence of gallstone as compared with general population [21, 22]. Acalovschi et al. demonstrated progressive impairment of gallbladder motility in the course of liver cirrhosis, suggesting that hypocontractility could promote gallstone formation in the advanced stage of the disease [5].

Our data suggest that gallbladder wall thickening and impairment of contractility occur early in the course of liver cirrhosis and precede the hemodynamic and clinical manifestations of portal hypertension. These changes seem to be partially related to the presence of hypoalbuminemia and, in our opinion, could predispose to gallstone formation in later stages of the disease. Nevertheless, further studies are needed to clarify the mechanisms of functional gallbladder alterations and pathogenesis of gallbladder wall thickening in cirrhotic patients and to investigate the role of bile acids or drugs active on gallbladder motility in the prevention of gallstones formation. 

## Figures and Tables

**Table 1:** Demographic and clinical characteristics of cirrhotic patients and healthy controls.

	Healthy controls	Cirrhotic patients				
Etiology	—	Total	Viral	Alcoholic	Alcoholic/viral	Autoimmune
Subjects n (%)	20 (100%)	23 (100%)	16 (69.6%)	4 (17.4%)	2 (8.7%)	1 (4.3%)
Sex	10 M–10 F	13 M–10 F	7 M–9 F	4 M–0 F	2 M–0 F	0 M–1 F
Age (mean ± SD)	53.6 ± 6.2	54.6 ± 8.7	53.7 ± 8.8	52.2 ± 9.2	60.5 ± 3.5	66
Albumin g/dL (mean ± SD)	3.6 ± 0.3	3.0 ± 0.3	3.0 ± 0.1	3.2 ± 0.5	2.9 ± 0.2	3.1

**Table 2:** Portal US and doppler parameters in cirrhotic patients and healthy controls.

Parameter	Cirrhotics (mean ± SD)	Controls (mean ± SD)	*P*
PD (mm)	8.42 ± 0.8	8.01 ± 0.7	.1
CSA (mm^2^)	55.7 ± 11.6	50.4 ± 9.7	.1
PV (cm/sec)	11.6 ± 1.6	12.6 ± 1.5	.06
PVF (mL/min)	652.1 ± 157.1	642.4 ± 149.9	.84

PD: portal vein diameter; CSA: cross-sectional area of portal vein; PV: mean portal velocity; PVF: portal vein flow.

**Table 3:** Gallbladder US parameters in cirrhotic patients and healthy controls.

US parameter	Cirrhotics (mean ± SD)	Controls (mean ± SD)	*P*
GWT (cm)	0.60 ± 0.22	0.21 ± 0.06	<.0001
FV (cm^3^)	37.8 ± 3.7	21.9 ± 4.2	<.0001
RV (cm^3^)	21.8 ± 3.02	4.6 ± 2.2	<.0001
%E	42.6 ± 7.8	80.3 ± 7.2	<.0001

GWT: gallbladder wall thickness; FV: fasting volume; RV: residual volume; %E: maximum percentage of emptying.
